# Supplementary material for: Peripheral myeloid-derived suppressor cells are good biomarkers of the efficacy of fingolimod in multiple sclerosis
Source: J Neuroinflammation. 2022 Nov 19;19:277. doi: 10.1186/s12974-022-02635-3 (PMC9675277; doi:10.1186/s12974-022-02635-3)
Supplement: Supplementary file 1 — Additional file 1: Table S1. List of antibodies used for flow cytometry and histology. [file 12974_2022_2635_MOESM1_ESM.docx]

| **Use** | | **Antibody** | **Target** | **Tissue/cells** | **Dilution^a^** | **Class** | **Clone** | **Manufacturer** | **Antibody ID** |
| --- | --- | --- | --- | --- | --- | --- | --- | --- | --- |
| **Flow cytometry** | Mouse | CD11b-PerCP Cy5.5 | Myeloid cells | Peripheral Blood/ Splenocytes | 0.2µg | Rat monoclonal | M1/70 | BD Biosciences | AB_394002 |
|  |  | CD11c-APC | Dendritic cells |  | 0.2µg | Hamster monoclonal | N418 | eBioscience | AB_469346 |
|  |  | F4/80-eFluor450 | Macrophages |  | 0.2µg | Rat monoclonal | BM8 | eBioscience | AB_1548747 |
|  |  | MHC-II-PE-Cy7.7 | Antigen presenting cells |  | 0.2µg | Rat monoclonal | M5/114.15.2 | eBioscience | AB_10870792 |
|  |  | Ly-6C-FITC | MDSCs |  | 0.2µg | Rat monoclonal | Ly-6C:AL-21 | BD Biosciences | AB_394628 |
|  |  | Ly-6G-PE^-^ |  |  | 0.2µg | Rat monoclonal | Ly-6C:1A8 | BD Biosciences | AB_394208 |
|  |  | CD3-PB | T cells |  | 0.2µg | Hamster monoclonal | 500A2 | BD Biosciences | AB_397063 |
|  |  | CD4-PE | CD4^+^-T cells |  | 0.1µg | Rat monoclonal | RM4-5 | BD Biosciences | AB_394585 |
|  |  | CD8-FITC | CD8^+^-T cells |  | 0.25µg | Rat monoclonal | 53-6.7 | BD Biosciences | AB_394568 |
|  |  | CD25-PE-Cy5.5 | Early activated T cells |  | 0.2µg | Rat monoclonal | PC61.5 | eBioscience | AB_11218898 |
|  |  | CD69-APC | Early activated T cells |  | 0.2µg | Hamster monoclonal | H1.2F3 | eBioscience | AB_1210795 |
|  | Human | CD11b-PE-Cy7 | MDSCs | Peripheral Blood | 0.25µg | Mouse monoclonal | ICRF44 | BD Biosciences | AB_396849 |
|  |  | CD33-APC |  |  | 0.2µg |  | WM53 | BD Biosciences | AB_398502 |
|  |  | HLA-DR-BV421 |  |  | 0.2µg |  | BM8 | BD Biosciences | AB_2687421 |
|  |  | CD14-PerCP-Cy5.5 |  |  | 0.25µg |  | MΦPg | BD Biosciences | AB_2737726 |
|  |  | CD15-FITC |  |  | 0.2µg |  | HI98 | BD Biosciences | AB_395801 |
| **Histology** | Human/mouse | NF-H | Non-phosphorylated neurofilament protein | Spinal cord | 1:200 | Mouse monoclonal | SMI-32 | Biolegend | AB_2564642 |

**Additional file 1: Table S1.** List of antibodies used for flow cytometry and histology

^a^Refers to the amount per million cells. Abbreviations: FITC, fluorescein isothiocyanate; PB, pacific blue; PE, phycoerythrin.
