# Supplementary material for: Peripheral myeloid-derived suppressor cells are good biomarkers of the efficacy of fingolimod in multiple sclerosis
Source: J Neuroinflammation. 2022 Nov 19;19:277. doi: 10.1186/s12974-022-02635-3 (PMC9675277; doi:10.1186/s12974-022-02635-3)
Supplement: Supplementary file 2 — Additional file 2: Table S2. Demographic data from the fingolimod patients included in the study. [file 12974_2022_2635_MOESM2_ESM.docx]

**Additional file 2: Table S2.** Demographic data from the fingolimod patients included in the study.

|  | **Total cohort**  **(N = 31)** | **R-MS**  **(N = 25)** | **NR-MS**  **(N = 6)** | **Ntz patients**  **(N = 11)** | **Non-Ntz patients**  **(N = 20)** |
| --- | --- | --- | --- | --- | --- |
| ***Age (years) ^¥^*** | 39.5 ± 1.31 | 40.3 ± 1.37 | 36.3 ± 3.67 | 41.0 ± 2.31 | 38.8 ± 1.61 |
| ***Sex (% of female)^$^*** | 26 (83.87%) | 21 (84%) | 5 (83.33%) | 9 (81.82%) | 17 (85%) |
| ***Previous DMTs*** | 1 naïve  15 IFNb  (5 IFNb-1b, 10 IFNb-1a)  4 GA  11 Ntz | 1 naïve  14 IFNb  (4 IFNb-1b, 10 IFNb-1a)  4 GA  6 Ntz | 1 IFNb-1b  5 Ntz | - | 1 naïve  15 IFNb  (5 IFNb-1b, 10 IFNb-1a)  4 GA |
| ***0 treatment*** | 1 | 1 | 0 | 0 | 1 |
| ***1 treatment*** | 12 | 9 | 3 | 2 | 10 |
| ***2 treatment*** | 4 | 4 | 0 | 0 | 4 |
| ***≥3 treatment*** | 14 | 11 | 3 | 9 | 5 |
| ***Basal ARR ^¥^,˜*** | 1.09 ± 0.18 | 1.24 ± 0.19 | 0.50 ± 0.34 | 0.27 ± 0.14 | 1.55 ± 0.20 * |
| ***Basal EDSS ^//^*** | 3.0 (2.0-4.0) | 3.0 (2.0-4.13) | 3.0 (2.5-4.0) | 3.0 (2.13-4.0) | 3.0 (2.0-4.25) |
| ***Gd+ lesions ^¥^*** | 1.45 ± 0.54 | 1.64 ±0.66 | 0.67 ± 0.422 | 0.18 ± 0.18 | 2.15 ± 0.80* |

*Abbreviations: ARR, Annualized Relapse Rate; GA, Glatiramer acetate; IFN, Interferon; NR-MS, Non-responders MS patients; Ntz, Natalizumab; R-MS, Responder MS patients;*

*^¥^ The values are the mean ± SEM of each group.*

*^//^ The values are the median IQR.*

*˜ For basal ARR, only the last year was considered.*

** Significant differences after Mann-Whitney test versus Ntz.*

*^$^ A Fisher exact test was used to compare the proportions.*
