# Supplementary material for: Peripheral myeloid-derived suppressor cells are good biomarkers of the efficacy of fingolimod in multiple sclerosis
Source: J Neuroinflammation. 2022 Nov 19;19:277. doi: 10.1186/s12974-022-02635-3 (PMC9675277; doi:10.1186/s12974-022-02635-3)
Supplement: Supplementary file 3 — Additional file 3: Table S3. Correlations between M-MDSCs and clinical parameters after 12 months of fingolimod treatment. [file 12974_2022_2635_MOESM3_ESM.docx]

|  | **EDSS** | **∆EDSS** | **New T2 lesions** | **New Gd^+^ lesions** | **Number of Relapses** | **ΔRelapses** |
| --- | --- | --- | --- | --- | --- | --- |
| **Total cohort _(N =31)_** | r = -0.085  p = 0.648 | r = -0.201  p = 0.276 | r = -0.137  p = 0.459 | r = -0.081  p = 0.664 | r = -0.115  p = 0.534 | r = -0.262  p = 0.153 |
| **R-MS_NEDA-3 (N = 13)_** | r = 0.149  p = 0.616 | r = 0.190  p = 0.516 | - | - | - | r = 0.129  p = 0.669 |
| **R-MS_CR_ _(N = 20)_** | r = 0.108  p = 0.644 | r = 0.098  p = 0.676 | r = 0.151  p = 0.517 | r = 0.107  p = 0.649 | - | r = -0.092  p = 0.695 |
| **R-MS _(N = 25)_** | r = 0.043  p = 0.835 | r = 0.091  p = 0.660 | r = 0.107  p = 0.607 | r = 0.095  p = 0.649 | r = 0.167  p = 0.423 | r = -0.010  p = 0.960 |

**Additional file 3: Table S3**. Correlations between M-MDSCs and clinical parameters after 12 months of fingolimod treatment.

*A Spearman’s correlation was carried out.*
